# Round the Hospitals

**Published:** 1919-03-01

**Authors:** 


### ROUND THE HOSPITALS.

The resignation of Dame Ethel Hope Becher, G.B.E., R.R.C., from her post as Matron-in-Chief of Queen Alexandra's Imperial Military Nursing Service marks definitely the transition from war conditions. It is well known that she consented to retain her office "for the duration" only at the call of duty, delaying her well-earned rest that she might not in the great emergency create the difficulties which a change of direction inevitably entails. Her powers were exactly what were needed in the crisis, and throughout the entire period of unexampled stress and strain on the nursing service of which she was chief, she has been a source of strength to all, a reserve force the value of which cannot be estimated. The *Times* says of her that "she possesses the gift of getting the best out of those who serve under her," and this is characteristic always of the highest products of our nursing system. She had, moreover, we quote again, "a keen insight into character, a remarkable memory and a capacity for instant decision." Such were precisely the qualities needed in war time. Dame Becher was one of the great matrons trained by Miss Luckes, under whom she served for six years. She obtained her Royal Red Cross, to which a bar was added last year, for services in the South African war, during which she learned much which was of immeasurable value to her, both in regard to nursing failures and the essentials for success in military nursing. She obtained the appointment of principal matron in 1903, and was promoted to be Matron-in-Chief seven years later.

---

The Central Council for District Nursing in London, at its annual meeting on Tuesday, passed a resolution expressing the opinion that the salaries of district nurses should be increased. The Executive Committee now have the matter under consideration, fortified by an instruction to take such action as might be necessary to secure such increase. The Committee is also empowered to inquire as to the adequacy and efficiency of the provision for district nursing in the County of London, and to make recommendations to the Council.

---

The report of the Central Midwives Board which has just been issued states that the Board has given its general approval to the proposals for a State-aided midwifery service in England and Wales formulated by the Association for Promoting the Training and Supply of Midwives. This is a very important announcement, and is likely to revolutionise the entire midwifery service which is at present being almost starved out of existence.

---

Hospital authorities in London have begun to give their adhesion to the principle of one day in seven. Miss Tice has signalised her appointment as Lady Superintendent at the Charing Cross Hospital by procuring this benefit for her nurses, and we understand is already satisfied that it makes for improved health and contentment. The St. Mary's Hospital for Women and Children at Plaistow has likewise conceded one day in seven to its nurses, and is taking over cottages in which to house the necessary additions to the staff. There is a growing consensus of opinion that the day off once a week is not only more serviceable to the nurse but also less upsetting to the hospital than the enforcement of an eight-hour day. The latter arrangement necessitates three shifts of nurses, and since during each period of eight hours one good meal must be partaken of outside the wards it reduces the working hours in practice to about seven in the day. If this plan be combined with the concession of one day off a week it means that the nurses' week would cover only forty-two hours of work. Nothing will be gained by pressing for concessions of this extreme nature.

---

At the conference on nurses' hours held under the auspices of the National Council of Women last week one speaker vehemently announced that all nurses' homes must be abolished, that an eight-hour day must be instituted, and that the nurses must be free to leave the institution at the conclusion of their eight-hour shift to return home. The fallacy of this contention has been often exposed, and it was hardly worth while to summon a conclave of matrons to listen to such nonsense. Lay people may think it sounds very attractive to leave the hospital at 4 P.M. and return home to freedom. But what about the staffs of hospitals which are not situated in a residential neighbourhood? And what about the plight of the woman who goes off duty at midnight after the trains and trams have stopped running? She must take perhaps an hour's walk to her lodging, let herself in to a dark house with a latch-key, and dispense with hot bath or hot meal. The multiple wretchednesses of lodgings compared with the appointments of the despised nurses' home would break down the physique of the strongest woman in a month or two. The impediments to day-sleep, even in an ordinary well ordered private house, have been discovered by many a V.A.D. worker. In lodging-houses the attempt to get rest would be altogether futile. The proposal to abolish the nurses' home comes from the ill-instructed people who are accustomed to rant against the "tyranny" of matrons, and is not supported by any who understand the facts.

---

Great developments in Red Cross work are under consideration at Devonshire House. The immense army of voluntary-aid workers is most unwilling to be demobilised. Thousands of women have tasted for the first time the real value and joy of work for others in connection with their hospital experiences. A vast proportion of these are part-time workers living in their own homes with domestic and other claims on their time. They cannot, if they would, devote themselves to hospital work as nurses. But they claim to offer their services to

**ROUND THE HOSPITALS—(*continued*).**

the aged, the defective, the erring, and the young. Could an efficient organisation be devised for teaching them to be of use under peace conditions an immense constructive force would be placed at the service of the country. We do not want amateur nurses, amateur health visitors, amateur welfare workers. But we can do with an almost unlimited supply of part-time mothers' helps and understudies to district nurses. We need women to devote themselves more and more under nurses' direction to the care of those long and difficult cases of tuberculosis and other chronic complaints for which room in institutions is not available. Everything depends on the quality of the teaching which will be given to the V.A.D. worker in the transition from war to peace, and we await the new developments in confidence.

QUEEN MARY'S Hostels for Nurses, first established in July 1915, are in great request during these days of demobilisation. They have met a need which will not entirely disappear when demobilisation is complete. For there are always a good number of military and naval nurses going out or coming home in the normal way, and for a very long time there are likely to be more than usual. At the first hostel in Bedford Place, directed from the beginning by Mrs. Kerr Lawson, O.B.E., over 9,000 ladies have stayed. Mrs. Lawson may be said to have raised the hostel system on to a new plane. From a more or less uncomfortable lodging for nurses perpetually passing on, she converted her hostel to a place of true refreshment both to mind and body, and her influence was immense over her ever-changing household. Her work is now accomplished, and she has handed it over to others, but her spirit remains in the houses she organised. The second hostel—in Russell Square—has received over 2,000 nurse guests; and the third—in Warwick Square—for nurses spending one night only in London, has provided accommodation for 4,000. Hundreds of nurses who needed a longer rest have been received at the hostels at Lady Desborough's beautiful riverside place, Taplow Court, for a fortnight. The Queen's interest in her hostels has been displayed in many directions.

THE new Secretary of the Scottish Board of the College of Nursing is Miss A. G. Pike, at present Night Superintendent of the Edinburgh Royal Infirmary. She has had very good experience in office work, having held the post of assistant to Miss Amy Hughes for some time at the Queen Victoria Jubilee Institute for Nurses in London. It appears to be a very happy choice on the part of the Scottish Board, and we have no doubt its affairs will continue to prosper under Miss Pike as they did under Miss Brunton, on whom fell the arduous labour of organising the office and its staff.

THE KING held an Investiture in the Ball-room of Buckingham Palace at 11 o'clock on Saturday, February 22. His Majesty personally conferred the following decorations on members of the Nursing Services: Royal Red Cross, First Class—Sister Cecilia Evans, Q.A.I.M.N.S. (R.); Sister Heila Bester and Sister Mildred Fynn, both of the South African M.N.S. Royal Red Cross, Second Class—Sister Annie Murray and Sister Evelyn Stubbington, both of the St. John Ambulance Brigade; Sister Mabel Lindsay and Sister Elizabeth Pierce, both of the Canadian Army N.S.; Sister Constance Ebden of the South African M.N.S. Also Mrs. Joan Kennard, Mrs. Eleanor Michelmore, Miss Elsie Moseley, and Miss Emma Whitehead, V.A.D. His Majesty conferred the Military Medal for bravery in the field on Sister Eileen King, Q.A.I.M.N.S. (R), and on Assistant Matron Mabel Chittock, Sister Margaret Balance, Sister Jane Bemrose, Sister Molly McGinnes, and Sister Katharine Warner, all of the St. John Ambulance Brigade. Queen Alexandra received the members of the Military Nursing Services who had been decorated by the King with the Royal Red Cross at Marlborough House after the investiture.

THE unintelligent abuse of lay managers at present prevalent appears to be founded on complete ignorance of the proper functions of the chairman of a voluntary hospital. He is the interpreter of the institution's needs to the public. Were he a doctor he might be considered biased in his estimates, a faddist or advertiser. He stands for his locality in a position not dissimilar from that of the War Minister in the House of Commons, who is rarely a soldier himself, but has to convey to Parliament the estimates furnished by the experts, having first formed his own judgment as to whether those estimates are reasonable. The chairman or treasurer, supported by the board, is responsible for finding funds by aid of which the schemes of the experts, whether medical or nursing, may be carried through. At the present juncture nursing reforms in hospitals may be said to hinge on the ability of doctors and matrons to show good cause for the improvements and extensions they advocate, but this is not a hardship, it is a protection. An appeal to the public for funds comes with irresistible force when it is backed by business men who, after devoting their attention to the proposed scheme, express themselves convinced of its necessity. To the lasting honour of our lay managers in this country it happens continually that the first movement towards reform is often made on their initiative.

AT a dinner given in Paris to Press representatives on February 21, the plans of the Red Cross Societies of Great Britain, the United States, France, Italy, and Japan for reconstruction work after peace has been declared were discussed. The two principal speakers were Sir Arthur Lawley, B.R.C.S., and Mr. Henry P. E. Davison, of the American R.C.S. An International Committee has already been appointed representing the above five countries, and a Convention has been arranged to take place in Geneva thirty days after peace is concluded, to which all the Red Cross organisations of the world

**ROUND THE HOSPITALS**—(*continued*).

will be invited to send delegates. Sir Arthur Lawley alluded to the wonderful spirit of self-sacrifice which has permeated every section of society during the war, and declared it would be deplorable if all these engines powerful for good were to be flung upon the scrap-heap or allowed to tumble to bits from rust or disuse. It is as much beyond the power of the little band of trained nurses to cope unaided with the enemies of mankind—famine, disease, and ignorance—which now menace civilisation, as it was for them to succour unaided the terrible flood of injured humanity which war poured forth. For every nurse trained to assume command and responsibility we need a dozen aids, capable of doing good service under skilled direction. There can be little doubt these aides will be forthcoming.

Masks and goggles have come to stay in hospitals receiving influenza patients. A beginning has at any rate been made in regard to the protection of the nurses. So long as matters were allowed fatalistically to run their course, every institution in which a case of influenza occurred or was received was liable to see the staff knocked over one after another like nine-pins, until only those with most resistance remained on duty, a sturdy but sorely tried little group, prone to envy sometimes their compeers safely tucked up in bed. All is changed now that the fact is established of the purely infective character of the disease. With elementary precautions influenza infection is no more likely to spread than typhoid. Wherever it does spread an inquiry should at once be held into the circumstances with a view to stopping the neglect of proper measures. The prevention of infection is well within the compass of medical and nursing knowledge, and wherever this fact is recognised the staff and patients alike are protected.

Although not seriously ill, we regret to learn that Dame Sidney Browne, who was taking a well-earned rest in the south of France, is still confined to her bed. The latest reports, however, are quite satisfactory. Her place in Pall Mall is being temporarily filled by Miss M. S. Riddell, R.R.C.

The Central Midwives Board for Scotland held its examination on January 27 simultaneously in three centres, the successful candidates numbering: Edinburgh 14, Glasgow 31, and Dundee 5, a total of 50.

The veil is being lifted here and there which has surrounded the doings of nurses in the war, and isolated examples of well-sustained effort are coming into evidence. Such a one is the story of Mrs. Carter, serving under the Joint Committee. She was in Malta with her husband when war broke out, and resumed her nursing career at the military Hospital there. Subsequently she was transferred from Valetta to France and served in the 14th General Hospital at Wimereaux and at Havre. She was in the terrible raid at Etaples in May, 1917. It began at the moment when all the nurses were hard at work with constant convoys of wounded arriving, and when the bombs began to fall there was great commotion. One fell right in the centre of the hospital and blew the huts to pieces. A large number of the patients were killed and some of the nursing sisters also. Mrs. Carter was struck by fragments and severely injured about the neck, shoulders, head and arms. She became unconscious and was carried out, but up till that moment continued with other sisters the work of removing the patients to comparative safety. She relates their reluctance to be moved while the nurses were still in danger; many could only be persuaded to go when they found no nurses would leave while any patients remained in danger.

Midwifery training at the Walton Institution of the West Derby Union has reached a high level, not a single failure to pass the examination of the C.M.B. having taken place during the last three years, in which thirty candidates have been placed on the register. These uniform successes reflect high credit on the senior medical officer, Dr. H. H. MacWilliam, and on the matron, Mrs. Roberts, indeed, on all who have assisted them.

The "Modern Hospital" (Chicago) good humouredly falls foul of us for having enunciated somewhere the truism "routine is not training," and contends that "some parts of a nurse's duties are too important to be exposed to the hazards of the wayward processes of thought." It may be true that nurses ought to take certain precautions "as a part of an absolutely unvarying routine, requiring no effort of the brain or will," but if the routine be unintelligent sooner or later the mind rebels against it and the habit is lost. The essence of good training is to set brain and will to dominate the muscles until actions become both intelligent and automatic.

Best congratulations to Manchester on the success of the Victory Ball. We understand it has resulted in a net profit of £3,848, a wonderful profit on one night's dancing, which speaks to capital organisation on the part of the *Daily Sketch*. Half the sum goes to the local branch of the College of Nursing, and the remainder to the Nation's Fund for Nurses.

**☛ It is our invariable practice to pay for all items of news which we may publish. The minimum payment is 5s. Paragraphs of about twenty lines in length—that is to say, of 150 words or less—are preferred. Contributions should be addressed to The Editor, The Hospital, 28 and 29 Southampton Street, Strand, London, W.C. 2, and, to be in time for the current week's issue, should reach him by the first post on Mondays.**

